# The complete chloroplast genome of *Primula helodoxa*, a species endemic to China

**DOI:** 10.1080/23802359.2019.1698988

**Published:** 2019-12-12

**Authors:** Li Zhang, Xiong Chen, Yuan Huang, Zhikun Wu

**Affiliations:** a School of Life Sciences, Yunnan Normal University, Kunming, Yunnan, PR China;; b Department of Pharmacy, Guizhou University of Traditional Chinese Medicine, Guiyang, PR China

**Keywords:** Complete chloroplast genome, *Primula helodoxa*, endemic species

## Abstract

*Primula helodoxa* Balf.f. is a species endemic to southeastern China distributed in Tengchong county of Yunnan Province. Here is the first time to report the complete chloroplast genome sequence of *P. helodoxa*. The complete chloroplast genome was 151,909 bp in length, containing a large single-copy (LSC) region of 83192 bp, a small single-copy (SSC) region of 17,797 bp, and a pair of inverted repeats (IRs) regions of 25,460 bp. There are 137 genes in total, 91 protein-coding genes, eight rRNA genes, and 38 transfer RNA genes. The phylogenetic tree showed relative relationship of *P. helodoxa* and *P. chrysochlora*.

*Primula helodoxa* Balf.f. is a species endemic to southeastern China distributed in Tengchong county of Yunnan Province (Hu and Kelso 1996). This species was published in 1916 as a new species in the *Sect. Proliferae* of family Primulaceae, then be treated as a member of group *Primula prolifera* Wallich (Richards 1993, 2003). In recent 20 years, *P. helodoxa* has been found again in Tengchong County since first discovery in 1910s and treated as an independent taxon in Flora of China (Hu and Kelso 1996). Because *P. helodoxa* has bright golden yellow corolla, it is a valuable parent to hybridize with relative candelabrum species. Here, we first report the complete chloroplast genome sequence of *P. helodoxa* for understanding its systematics and provide scientific basis for the breeding hybrid in the garden.

The fresh leaves of *P. helodoxa* were collected from Tengchong, Yunan Province. The voucher specimen was deposit in the Herbarium of Yunnan Normal University (accession no: WZK140621). Total genomic DNA was extracted from the isolated chloroplasts using a modified CTAB method. According to the criterion, we fragmented the DNA and used Illumina Hiseq X Ten sequencer to construct the genomic library for Illumina paired-end (PE) sequencing. Then, NOVOplasty v2.7.2 (Dierckxsens et al. 2017) has been used to assemble the complete chloroplast genome of *P. helodoxa*. We also used Geneious v 8.0.2 software to annotate the chloroplast genome assembled (Kearse et al. 2012).

The annotated complete chloroplast genome of *P. helodoxa* was 151,909 bp in length with an overall GC content 37.0% (GenBank number MN504640). The assembled genome contained a large single-copy (LSC) region of 83,192 bp, a small single-copy (SSC) region of 17,797 bp, and a pair of inverted repeats (IRs) regions of 25,460 bp. In total, the chloroplast genome sample consisted of 137 gene, 91 protein-coding genes, eight rRNA genes, and 38 transfer RNA genes, including 115 unique genes, 81 unique CDSs, 30 unique tRNAs, and four unique rRNAs.

The phylogenetic relationship between *P. helodoxa* and relative species of *Primula* was inferred base on the relative 11 species with complete chloroplast genomes downloaded from GenBank. All the sequences were aligned by the MAFFT version 7 software (Katoh et al. 2002). The maximum-likelihood (ML) tree was constructed using IQ-TREE v1.6.10 (Nguyen et al. 2015) and performed base on TVM + F+R2 model according to Bayesian information criterion using ModelFinder (Kalyaanamoorthy et al. 2017); ultrafast bootstrap (UFBoot) was used to test branch supports (Hoang et al. 2018) and SH-like approximate likelihood ratio (Figure 1). The phylogenetic tree showed that *P. helodoxa* and *P. chrysochlora* formed a monophyletic clade with 100% bootstrap value, being consistent with former taxonomic studies of the genus *Primula* (Yan et al. 2015). The complete chloroplast genome of *P. helodoxa* also provided a reference chloroplast genome for the breeding in garden as well as the phylogentic studies of Primulaceae.

**Figure 1.**
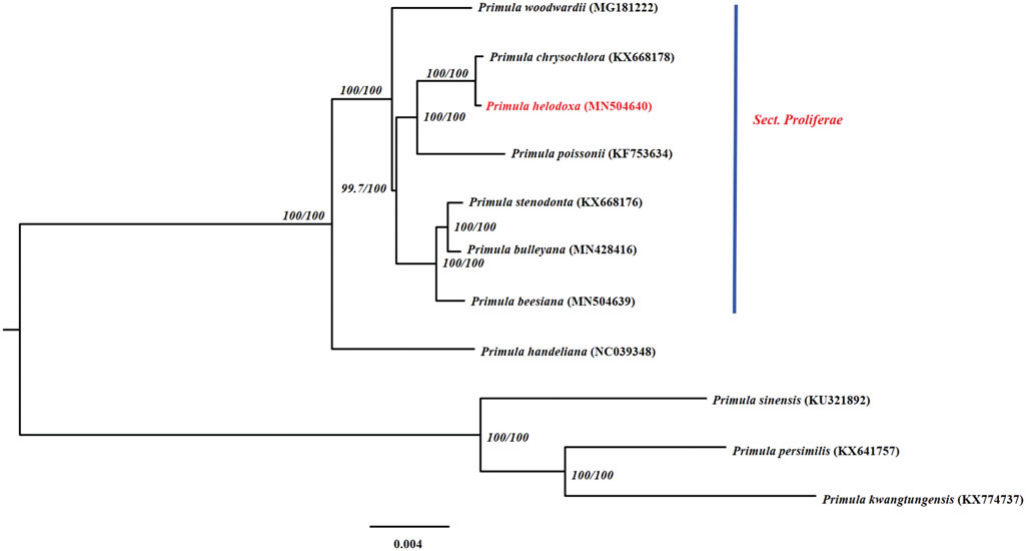
ML phylogenetic tree of *Primula helodoxa* and 10 Primulaceae species based on chloroplast complete genome, branch supports values were reported as SH-aLRT/UFBoot, green solid dot denotes supports values of 100/100.
